# Adolescents’ pain and distress during peripheral intravenous cannulation in a paediatric emergency setting

**DOI:** 10.1007/s00431-021-04169-x

**Published:** 2021-07-03

**Authors:** Giorgio Cozzi, Marta Cognigni, Riccardo Busatto, Veronica Grigoletto, Manuela Giangreco, Mariasole Conte, Egidio Barbi

**Affiliations:** 1 Institute for Maternal and Child Health, IRCCS Burlo Garofolo, Trieste, Italy; 2 University of Trieste, Trieste, Italy

**Keywords:** Adolescents, Emergency department, Distress, Needle phobia, Pain

## Abstract

The objective of the study is to investigate pain and distress experienced by a group of adolescents and children during peripheral intravenous cannulation in a paediatric emergency department. This cross-sectional study was performed between November 2019 and June 2020 at the paediatric emergency department of the Institute for Maternal and Child Health of Trieste, Italy. Eligible subjects were patients between 4 and 17 years old undergoing intravenous cannulation, split into three groups based on their age: adolescents (13–17 years), older children (8–12 years), and younger children (4–7 years). Procedural distress and pain scores were recorded through validated scales. Data on the use of topical anaesthesia, distraction techniques, and physical or verbal comfort during procedures were also collected. We recruited 136 patients: 63 adolescents, 48 older children, and 25 younger children. There was no statistically significant difference in the median self-reported procedural pain found in adolescents (4; IQR = 2–6) versus older and younger children (5; IQR = 2–8 and 6; IQR = 2–8, respectively). Furthermore, no significant difference was observed in the rate of distress between adolescents (79.4%), older (89.6%), and younger (92.0%) children. Adolescents received significantly fewer pain relief techniques.

*Conclusion:* This study shows that adolescents experience similar pain and pre-procedural distress as younger children during peripheral intravenous cannulation.

**Table:** 

**What is Known:** *• Topical and local anaesthesia, physical and verbal comfort, and distraction are useful interventions for pain and anxiety management during intravenous cannulation in paediatric settings. * *• No data is available on pain and distress experienced by adolescents in the specific setting of the emergency department.*	
**What is New:** *• Adolescents experienced high levels of pre-procedural distress in most cases and similar levels of pain and distress when compared to younger patients* *• The number of pain relief techniques employed during procedures was inversely proportional to patient’s age, topical or local anaesthesia were rarely used *

**What is Known:**

*• Topical and local anaesthesia, physical and verbal comfort, and distraction are useful interventions for pain and anxiety management during intravenous cannulation in paediatric settings. *

*• No data is available on pain and distress experienced by adolescents in the specific setting of the emergency department.*

**What is New:**

*• Adolescents experienced high levels of pre-procedural distress in most cases and similar levels of pain and distress when compared to younger patients*

*• The number of pain relief techniques employed during procedures was inversely proportional to patient’s age, topical or local anaesthesia were rarely used *

## Introduction

Needle-related procedures are part of routine medical care in emergency departments (EDs) and are the most commonly performed medical procedures among children and adolescents [3], representing a potential source of considerable pain and distress [3, 11]. The relationship between painful needle-procedure experiences and needle phobia is also well established [20, 22]. As a matter of fact, needle phobia has been identified as a relevant cause of vaccination refusal and delayed access to care in adults with measurable health related damages [20]. Clinical guidelines have been developed in order to improve the management of paediatric pain and ensure an appropriate use of pain and distress relief techniques during needle procedures [21, 9]. However, most of the available studies focus on patients between 3 and 12 years of age, and few data sources are available regarding pain and distress experienced by adolescents during these procedures. To the best of our knowledge, no data is available on pain and distress experienced by adolescents in the specific setting of the paediatric emergency department. In this context, adolescents are the older patients. They may show less behavioural and verbal signs of distress compared to younger patients, and this may lead operators to underestimate their distress which may result in a limited use of both pharmacological analgesia and distracting techniques [18].

The aim of this study is to investigate the pain and the distress experienced by a population of adolescents in a paediatric ED, compared to younger patients, and to investigate how the age of the participants involved correlates with the number of pain relief techniques employed, and with parents’ behaviour during the procedures.

## Methods

This was a single-centre cross-sectional study conducted between November 2019 and June 2020 at the paediatric emergency department of the tertiary level children’s hospital Institute for Maternal and Child Health of Trieste, Italy. The study received approval from the Institutional Review Board of the Institute (RC 25/2019). All of the children’s parents were provided written informed consent before participation. We enrolled patients from 4 to 17 years undergoing peripheral intravenous cannulation. Exclusion criteria included presence of cognitive impairment, decreased vigilance (GCS < 15), need for urgent treatment, need for sedation during the procedure, administration of analgesics in the previous 8 h, and insufficient understanding of the Italian language or inability of the parents to provide a written informed consent.

For every enrolled patient, the following features were collected: age, site of procedure, presence of a chronic disease, number of similar procedures in the last year, previous needle-related traumatic experiences, number of procedures performed by the operator in the last month, number of operators acting on the patient during procedure, type of techniques used for pain and distress management during procedures, need for physical restraint, and lastly, parents’ behaviour during the procedure.

Enrolled patients were divided into three groups according to their age: adolescents (13–17 years), older children (8–12 years), and younger children (4–7 years).

The procedural pain was self-reported by patients, immediately after the first attempt of peripheral intravenous cannulation, and was measured through the Faces Pain Scale-Revised (FPS-R), which includes a numeral rating scale from zero (no pain) to 10 (maximum pain), and a series of faces with an expression changing from no pain to severe pain [5]. Scores between 4 and 6 were considered to be moderate pain, and scores between 7 and 10 as severe pain.

The procedural distress was measured immediately before the procedure according to the patients’ age. In patients from 8 to 17 years old, it was self-reported through a validated visual scale named the “distress thermometer” [6], which uses a score from zero (no distress) to 10 (severe distress). In children from 4 to 7 years old, the distress level was measured by an external observer using the Children’s Emotional Manifestation Scale (CEMS scale) [10]. This scale considers facial expression, vocalizations, activity, interaction, and cooperation, with a score from 5 (no distress) to 25 (severe distress). To compare the results, both scales were then categorized in two classes: presence and absence of distress (with presence defined as a score higher than 0 in the “distress thermometer” and higher than 5 in CEMS scale). Significant distress was considered to be anything with a value higher than 3 on the thermometer and 10 on the CEMS scale.

For every procedure, we recorded the following pain relief strategies: (1) use of topical and local anaesthesia, (2) use of distraction techniques (songs, rhymes, storytelling, soap bubbles, music, cartoons, tablet computers, television), (3) use of physical comfort measures, and (4) verbal comfort measures adopted by caregiver or hospital staff during the procedure. Holding the patient on parents’ laps or holding their hand, touching or caressing the patient was considered physical comfort measures. On the contrary, the active intervention required to keep a resistant or agitated child still during procedures was considered as physical restraint. Talking to patients in order to comfort them was considered a verbal comfort measure.

All the study-related data was collected by an external observer in a data collection form specifically developed for the study. The external observer was a senior medical student, specifically trained on the study purpose. He did not take any active part in procedures apart from recording pain and distress scores, the number of pain and distress relief employed, and the actions of operators and parents. Trying to assure an unbiased picture of procedural pain and distress management, the operators were not aware that the external observer collected data related to the techniques employed and the staff behaviour as well.

The primary study outcome was the comparison of median self-reported procedural pain among the three groups of patients. Secondarily, the three groups of patients were compared in terms of presence of distress, relationship between distress and pain, and number and type of pain relief techniques employed during procedures.

### Statistical analysis

Descriptive analysis of all variables was performed by reporting frequencies and percentages for categorical, median, and interquartile ranges for continuous variables. Differences in the distribution of continuous variables between the three age groups were assessed with the Kruskal Wallis test, while the association between categorial variables and age group were tested by chi-square or the exact Fisher test, as appropriate. Wilcoxon Mann Whitney test was used to evaluate association between pre-procedural distress and self-reported procedural pain by age group. A *p* < 0.05 was considered as statistically significant. Statistical analyses were performed using SAS software, Version 9.4 (SAS Institute Inc., Cary, NC, USA).

## Results

During the study period, 164 patients were considered eligible for the study. Twenty-eight patients (17%) were excluded, due to the exclusion criteria previously outlined. We prospectively enrolled 136 patients aged 4–17 years. An overview of the patients’ and procedures’ characteristics is provided in Table 1. The proportion of patients affected by chronic diseases who underwent needle procedures in the last 12 months and who declared previous negative experiences during needle procedures was similar in the three groups of patients.

**Table 1:** Patients’ characteristics by age group

	Adolescents (13–17 years)	Older children (8–12 years)	Younger children (4–7 years)	p-value
Number of patients	63	48	25	
Sex, n (%)				
Female	42 (66.7)	22 (45.8)	10 (40.0)	
Affected by a chronic disease, n (%)	10 (15.9)	3 (6.3)	2 (8.0)	0.3^**^
Needle-related procedures in the last 12 months, median (IQR)	1 (0–2)	0 (0–1)	0 (0–1)	0.2^*^
Reported previous negative experiences with needle-related procedures, n (%)	7 (11.1)	2 (4.3)	1 (4.2)	0.4^**^
Number of procedures performed by the operator in the previous month, n (%)				
< 5	12 (19.0)	7 (14.6)	2 (8.0)	0.7^**^
5–10	2 (3.2)	1 (2.1)	0 (0.0)	
> 10	49 (77.8)	40 (83.3)	23 (92.0)	
Location, n (%)				
Forearm	4 (6.4)	1 (2.1)	0 (0.0)	0.01^**^
Cubital fossa	43 (68.3)	28 (58.3)	9 (36.0)	
Hand	16 (25.4)	19 (39.6)	16 (64.0)	
Number of operators during procedures, median (IQR)	2 (2–3)	2 (2–3)	2 (2–3)	0.1^*^
First attempt success, n (%)	57 (90.5)	38 (79.2)	22 (88.0)	0.2^**^

^*^Kruskal Wallis test

^**^Chi-square or Fisher test, as appropriate

Table 2 shows the main study results. Levels of self-reported pain (median (IQR)) were similar in adolescents (4 (2–6)), older (5 (2–8)), and younger children (6 (2–8)).

**Table 2:** Main study results

	Adolescents (13–17 years)	Older children (8–12 years)	Younger children (4–7 years)	p-value
Number of patients	63	48	25	
Pre-procedural distress, median (IQR)	5 (2–7) (range 0–10)	5 (3–9) (range 0–10)	16 (9–20) (range 5–25)	< .0001^*^
Pre-procedural distress, n (%)				0.2 ^**^
Yes	50 (79.4)	43 (89.6)	23 (92.0)	
No	13 (20.6)	5 (10.4)	2 (8.0)	
Severe pre-procedural distress, n (%)	45 (71.4)	39 (81.3)	18 (72.0)	0.5 ^**^
Self-reported procedural pain, median (IQR)	4 (2–6)	5 (2–8)	6 (2–8)	0.2 ^*^
Number of pain relief techniques used during procedures, median (IQR)	1 (1–2)	2 (1–3)	3 (2–4)	< .0001^*^
Type of relief techniques employed, n (%) Topical local anaesthesia Distraction Physical comfort Verbal comfort	2 (3.2) 35 (55.6) 14 (22.2) 46 (73.0)	21 (43.8) 30 (62.5) 14 (29.2) 36 (75)	20 (80.0) 18 (72.0) 12 (48.0) 20 (80.0)	< 0001^**^ 0.4^**^ 0.1^**^ 0.8^**^
Need for physical restraint, n %	0 (0.0)	7 (14.6)	5 (20.0%)	0.001

^*^Kruskal Wallis test

^**^Chi-square or Fisher test, as appropriate

Most patients reported or were observed to experience distress before the procedure: 50 adolescents (79.4%), 43 older (89.6%), and 23 younger (92%) children, with no statistically significant differences between age classes (*p* = 0.2) found. The rate of severe pre-procedural distress was also similar among age classes (*p* = 0.5).

Table 3 shows the association between pre-procedural distress and self-reported procedural pain by age group.

**Table 3:** Association between pre-procedural distress and self-reported pain by age group

		Self-reported procedural pain		
Age groups	Pre-procedural distress	n	median (IQR)	Wilcoxon Mann Whitney p-value
Adolescents	Yes	50	5 (3–7)	0.01
	No	13	1 (0–4)	
Older children	Yes	43	5 (3–8)	0.002
	No	5	0 (0–0)	
Younger children	Yes	23	7 (2–8)	0.4
	No	2	3.5 (2–5)	

A significant association between the presence of pre-procedural distress and higher scores of self-reported procedural pain (*p* = 0.01 and *p* = 0.02 respectively) was observed among adolescents and older children. This association was not significant among younger children (*p* = 0.4).

The number of pain relief techniques employed during procedures was inversely proportional to patient’s age (Table 2), and this relationship was statistically significant (*p* < 0.0001).

In detail, topical or local anaesthesia was used in 2 adolescents (3.2%), 21 older children (43.8%), and 20 younger children (80.0%) (*p* < 0.0001); distraction techniques were employed in 35 adolescents (55.6%), 30 older children (62.5%), and 18 younger children (72.0%) (*p* = 0.4); physical comfort was provided for 14 adolescents (22.2%), 14 older children (29.2%), and 12 younger children (48.0%) (*p* = 0.1); verbal comfort was reported in 46 adolescents (73.0%), 36 older children (75.0%), and 20 younger children (80.0%) (*p* = 0.8).

**Fig. 1:**
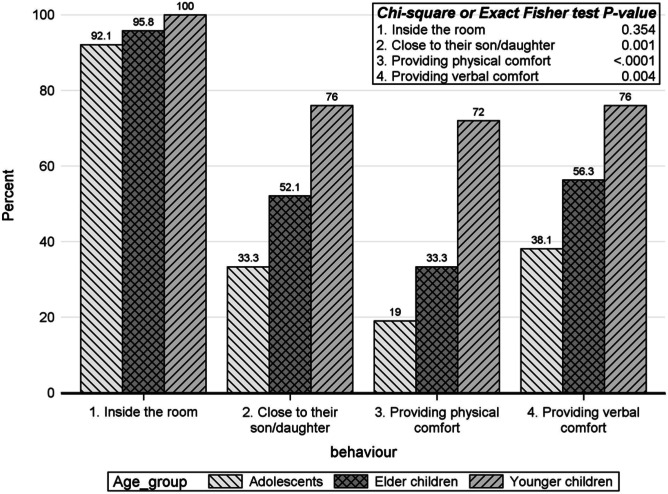
Parents’ behaviour during procedure by age group

Figure 1 shows parents’ behaviour during procedures. Adolescents received significantly less physical and verbal consolation from their parents. In addition, the parents were significantly less close to their adolescent sons/daughters during the procedure.

## Discussion

This study shows that adolescents experienced similar pain and pre-procedural distress to younger patients during peripheral intravenous cannulation. Adolescents received fewer pain relief techniques and did not receive topical anaesthesia in most of the cases.

Needle-related procedures are the most common sources of distress and pain in paediatric Eds [2]. While the concern for understanding and management of the unpleasant sensory and emotional experience associated with painful medical procedures in children has increased in past years [4, 8], limited data is available for adolescents. A recent Cochrane review on the psychological interventions used to reduce needle-related pain and distress considered 28 studies, but only one involving adolescents [1]. While adolescents give a psychological meaning of their pain and can describe it, they also tend to hide its expression due to the fear of not being seen as adults, which contributes to a negative experience [7]. Regarding the high prevalence of pre-procedure distress in our sample, these results confirm the findings of a previous study investigating needle-related pain in paediatric oncology patients [12] that reported a prevalence of distress of 75% [12]. The association between pre-procedure distress and procedural pain in children is well known [13], and the results of this study confirm that it applies to adolescents as well. In order to highlight the possible factors which could have influenced the study results, derived from the baseline conditions and the previous experiences of the patients, we collected data regarding the prevalence of chronic diseases, the number of procedures performed in the last 12 months and the number of previous negative experiences in the three groups of patients. We did not find significant differences.

Fewer pain and distress relief interventions were employed in adolescents when compared to younger children, highlighting a tendency to underestimate and under treat their pain and distress. Furthermore, adolescents generally received limited consolation by their parents, compared to older and younger children. Future studies need to address how the patients’ age may influence the behaviour of health care operators towards the use of pain and distress relief techniques in emergency settings.

Evidence shows that adolescent experiences may play a role in the genesis of needle phobia [7, 13, 17, 14]. Therefore, an awareness of the impact on needle procedures in the EDs may be relevant in order to develop strategies to limit needle phobia in adults and its related consequences and costs. Topical and local anaesthesia through the application of anaesthetic creams or injecting buffered lidocaine is the most effective pain relief technique during peripheral intravenous cannulation [2, 15]. Nevertheless, a recent survey focused on European paediatric EDs showed that their availability and use is still limited [19]. Notably in this study, only 3.2% percent of adolescents received EMLA cream compared to 43.8% of older children and 80% of young children. Topical and local anaesthesia and distraction techniques are effective and economic analgesic strategies. We suggest that they should be used more frequently when dealing with adolescents, even in the ED settings, and particularly in patients showing a high level of pre-procedural distress.

This study has some limitations. It is a single-centre experience with a limited sample size; therefore, the generalizability of results should be taken with caution. We were not able to find a statistically significant difference in median pain scores among adolescents and younger patients, but this could be related to the limited sample size. Remarkably, in this setting, a clinically relevant difference such as a difference perceived by the patient may be different from a statistically significant one [16]. As a matter of fact, in this study, the median difference between pain scores among adolescents and younger children seemed to reach a minimum clinically important difference favouring adolescents.

Procedural distress was measured only before the peripheral intravenous cannulation and not during or after procedures. The data collection form was developed specifically for the study and was not validated. Furthermore, this instrument was only quantitative and we did not perform a qualitative analysis of the pain and distress relief techniques employed. Child life specialists are not available in Italy and distraction techniques and comfort measures are managed by paediatric doctors and nurses who receive a dedicated training as part of their professional role. All the procedures were recorded by a single external observer specifically instructed in the recognition of pain and distress relief techniques. The observer was present in the room during the procedures and did not take any active part in them. Nevertheless, we cannot exclude that some factors may have influenced their judgment. Moreover, we cannot exclude that staff and parents’ behaviour may have been influenced by the participation in the study or by some cultural influence causing less use of pain and distress relief techniques in adolescents in this specific ED compared to other EDs in different regions.

Finally, a separate analysis of distress in patients receiving topical anaesthesia was not performed as only two patients in the adolescents group received EMLA cream.

The point of strength is the use of validated and standardized scales to measure pain and distress and the fact that a categorization was applied to allow the comparison between different ages.

In conclusion, this study, which was performed in a paediatric emergency setting, showed that adolescents experienced similar values of pain and distress during peripheral intravenous cannulation when compared to children, while receiving less pain relief techniques.

## Data Availability

Complete data and material are available from the corresponding author, upon reasonable request.

## Abbreviation

ED: Emergency Department
